# Language-Eloquent White Matter Pathway Tractography and the Course of Language Function in Glioma Patients

**DOI:** 10.3389/fonc.2018.00572

**Published:** 2018-12-06

**Authors:** Sebastian Ille, Lara Engel, Anna Kelm, Bernhard Meyer, Sandro M. Krieg

**Affiliations:** ^1^Department of Neurosurgery, Klinikum Rechts der Isar, Technische Universität München, Münich, Germany; ^2^TUM-Neuroimaging Center, Klinikum Rechts der Isar, Technische Universität München, Münich, Germany

**Keywords:** fiber tracking, glioma, language, nrTMS, tractography

## Abstract

**Object:** As various recent studies show, damage to white matter pathways leads to permanent functional deficits in a high percentage of patients. Particularly the subcortical language network is complex, and its visualization has a tremendous relevance for neurosurgeons. This pilot study aims to correlate language-eloquent white matter pathways with the course of language function after the resection of left-sided perisylvian gliomas.

**Methods:** We included 10 patients who underwent resection of highly language-eloquent high- (9 pts) and low-grade gliomas (1 pts). We performed navigated repetitive transcranial magnetic stimulation (nrTMS)-based tractography via diffusion tensor imaging fiber trackings (DTI FT) preoperatively (PRE-1), postoperatively (POST-1), and at long-term follow up or tumor recurrence (PRE-2). We separately tracked the inferior fronto-occipital fascicle (IFOF), the frontal aslant tract (FAT), and the superior longitudinal (SLF), and arcuate fascicle (AF), and correlated the amount of visualized fibers to the patients' language function at each date.

**Results:** The changes of nrTMS-based DTI FTs of single white matter pathways correlated with the according status of language function for any of the pathways in 80% of patients and in 19 of 30 (63%) single pathway comparisons between PRE-1 and POST-1. Between POST-1 and PRE-2 the nrTMS-based DTI FTs correlated with the status of language function for any of the pathways in all patients and in 24 of 30 (80%) single pathway comparisons. Single FT results correlated with the according status of language function at POST-1 in 60, 70, and 60% of cases, and with the according status of language function at PRE-2 in 60, 90, and 90% of cases for the tracking of the IFOF, FAT, and SLF/AF, respectively.

**Conclusion:** By the present results we were able to show that nrTMS-based DTI FT of the IFOF, FAT, and SLF/AF mainly correlates with the according status of language function preoperatively, postoperatively, and at long-term follow up after the resection of left-sided perisylvian gliomas.

## Introduction

Cortical and subcortical anatomy of human language function is complex. Decades after Broca, Wernicke, and Geschwind we know that cortical language function is highly individualized and only the superficial part of a complex network. As we have learned from studies examining cortical and subcortical representations of language function during the resection of language-eloquent gliomas by the gold standard technique direct electrical stimulation (DES) during awake surgery, large parts of the cortex are resectable without causing a functional deficit, while subcortical white matter pathways need to be preserved to a larger extent (1–3). Hence, preserving these structures is essential during the resection of eloquent brain tumors. Our current knowledge of the complex subcortical language network bases on white matter tractography by diffusion tensor imaging fiber tracking (DTI FT) and insights through DES during awake craniotomies (4–8). These techniques and anatomical studies led to the currently most accepted *dual stream model of language* (8, 9). The ventral stream passing through the external capsule (EC) and consisting of the inferior fronto-occipital fascicle (IFOF), the uncinate fascicle (UF), and the inferior longitudinal fascicle (ILF) carries semantic information and connects associated cortical areas of the frontal, temporal, parietal, and occipital lobe (8–10). The dorsal stream, which runs around the sylvian fissure connecting the frontal, temporal, and parietal perisylvian cortices, includes the arcuate fascicle (AF) and parts of the superior longitudinal fascicle (SLF) and transmits phonological processing and articulation (8, 9). Apart from this long-connectivity language network, recently published studies confirmed the language-eloquent role of the frontal aslant tract (FAT) connecting the supplemental motor areas (SMA) and pre-SMA with Broca's region (11–14).

Meanwhile, language mapping by navigated repetitive transcranial magnetic stimulation (nrTMS) has emerged to a commonly used preoperative mapping technique (15–18). It enables to perform a cortical mapping by inducing *virtual lesions* non-invasively and is thereby based on the same principle of functioning as the gold standard technique DES during awake craniotomy (19). Also the feasibility of visualizing subcortical white matter pathways by nrTMS-based DTI FT has repeatedly been shown (20, 21). Hence, the approach of choosing function-based regions of interest (ROI) in terms of a cortical nrTMS language mapping enables to select single white matter pathway tractographies. Additionally, the non-invasive mapping technique provides the opportunity to perform function-based DTI FTs during long-term follow up without tumor recurrence.

The present study evaluates if we are able to perform single white matter pathway tractographies of the FAT, the IFOF, and the SLF/AF as the core of white matter language pathways by nrTMS-based DTI FT in patients suffering from highly language-eloquent gliomas. Furthermore, the study examines if we can visualize the changes of the extension of the subcortical language network in dependence on the status of language function preoperatively, postoperatively, and at long-term follow up.

## Materials and Methods

### Ethics

The experimental setup was approved by our local ethics committee (registration number: 222/14) and was conducted in accordance with the Declaration of Helsinki. Written informed consent was obtained from all patients prior to the examination.

### Patients

For the present study we included patients with left-sided language-eloquent gliomas of our prospective language mapping cohort. In order to perform nrTMS-based DTI FT preoperatively, postoperatively, and at long-term follow up, patients had to meet the inclusion criteria of repeated nrTMS language mappings and DTI sequences. Patients with an age younger than 18 years, general TMS exclusion criteria, such as cochlear implants or a cardiac pacemaker, or a too severe aphasia (<60% properly named pictures during baseline object naming) were excluded from the study (22, 23).

### Language Assessment

For the present analysis we evaluated the patients' neurological statuses including an aphasia grading adapted from the Aachener Aphasia Test (AAT) (24) at no fewer than six points in time: before surgery 1 (PRE-1), 5 days after surgery 1 (POD5-1), 3 months after surgery 1 (POM3-1), before surgery 2 or during long-term standard follow-up in case of no tumor recurrence (PRE-2), 5 days after surgery 2 (POD5-2), and 3 months after surgery 2 (POM3-2). We graded aphasias from 0 to 3 (0 = no impairment of language function; 1 = slight impairment of daily communication; 2 = moderate impairment of language function, daily communication possible; 3 = severe impairment of language function, daily communication not possible), and in case of an aphasia by the addition of an A (non-fluent) or B (fluent) (15–18, 20, 24, 25).

### Magnetic Resonance Imaging

The sequences were performed on 3 T magnetic resonance scanners (Philips Medical System, Netherlands B.V.). All patients obtained MRIs according to the standard glioma protocol at our department including a T1-weighted three-dimensional (3-D) gradient echo sequence with intravenous contrast administration for anatomical co-registration, a T2-weighted 3-D FLAIR sequence, and DTI sequences with 6-32 orthogonal sequences. T1- and T2-weighted 3-D images were performed at each of the six points in time at which language performance was evaluated. We used the postoperative diffusion images at POD5-1 to evaluate subcortical ischemia. DTI sequences for nrTMS-based DTI FTs were conducted at PRE-1, PRE-2, and at one point in time between PRE-1 and PRE-2 (POST-1).

### Operative Technique

All surgeries were performed with a function-based approach for the resection of language-eloquent gliomas. Standardly, we used a neuronavigation system for all surgeries (Brainlab Curve, Brainlab AG, Munich, Germany). The neuronavigation system was used for craniotomy planning and intraoperative navigation. Additionally, preoperative nrTMS language mapping results and nrTMS-based DTI FTs were transferred to the neuronavigation system in all cases and thereby displayed on the navigation screen during the microsurgical resection. In cases of an additional language mapping by DES during awake craniotomy, the intraoperative mapping was guided by preoperative nrTMS language mapping data (26).

Within the evaluated period we performed eight language mappings by DES during awake craniotomy in the included patients. We therefore used our standard protocol with an asleep-awake-asleep approach and according to the guidelines for awake craniotomies (27–29). In these cases we used a combination of epinephrine and bupivacaine for regional anesthesia of the galea and dura, and a total intravenous anesthesia by remifentanil and propofol, which was stopped prior to intraoperative language mapping. Here, we performed an object naming task (ON) consisting of the pictures which were used for the ON during preoperative nrTMS language mapping. In order to detect intraoperative seizures we recorded a surface electroencephalogram. Cortical stimulation was performed with a bipolar electrode and subcortical stimulation was performed with a monopolar electrode (Inomed Medizintechnik GmbH, Emmendingen, Germany). After the language mapping phase the resection was performed under continuous monitoring of overt speech.

### Setup

#### nrTMS Language Mapping and nrTMS-Based DTI FT

We performed nrTMS language mappings using the eXimia nTMS system version 4.3 and a NEXSPEECH® module (Nexstim Plc, Helsinki, Finland) and an ON at PRE-1 and PRE-2 according to the recently published nTMS working group protocol (23). Afterwards, we exported the left-sided language-positive sites in terms of nrTMS in order to perform nrTMS-based DTI FT.

**Figure 1:**
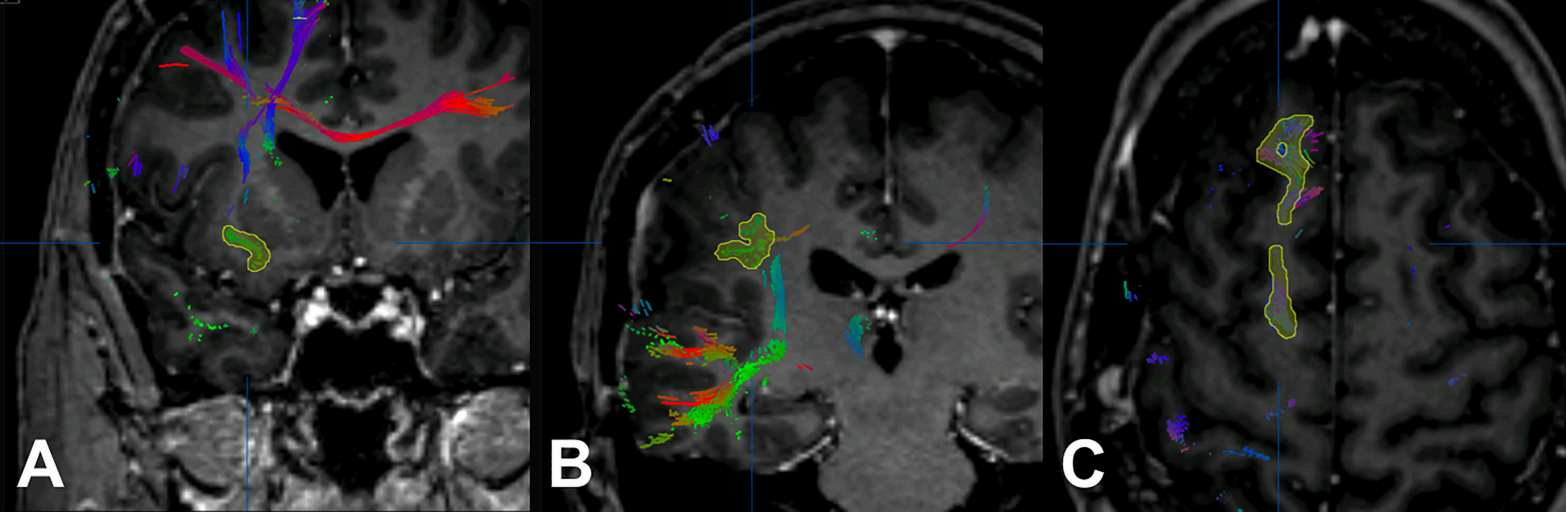
Additional regions of interest. The figure shows the process of adding further regions of interest for the inferior fronto-occipital fascicle (IFOF; **A**), the superior longitudinal fascicle and arcuate fascicle (SLF/AF; **B**), and the frontal aslant tract (FAT; **C**).

**Figure 2:**
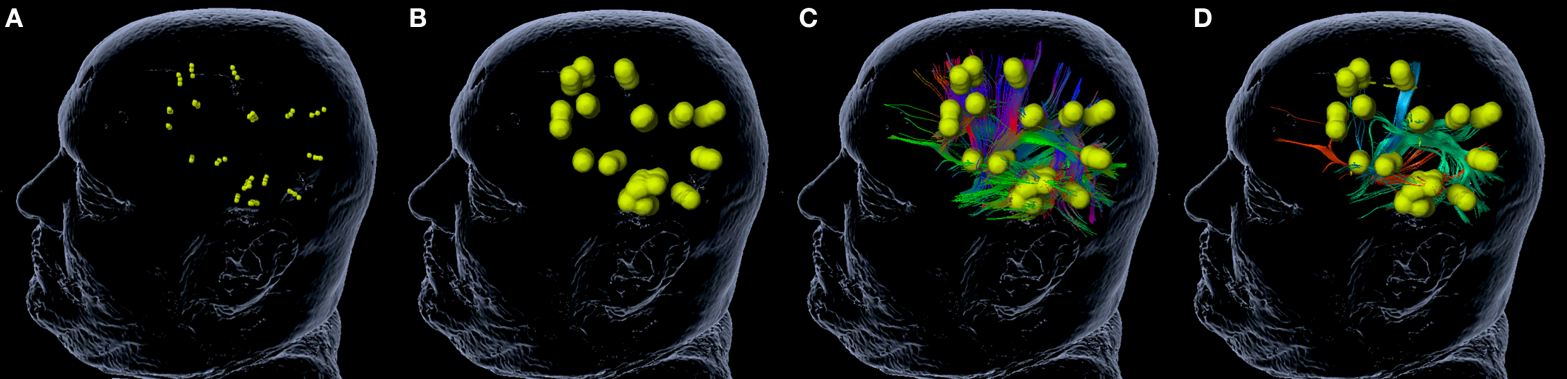
Tractography of single white matter pathways. The figure shows the process of tractography for single white matter pathways. A rim of 5 mm **(B)** is added to language-positive sites in terms of nrTMS **(A)**. The software calculates a whole-language network tractography **(C)**. By the addition of further regions of interest (ROI, Figure 1) the whole-language network tractography can be reduced to the inferior fronto-occipital fascicle (IFOF; red), the superior longitudinal fascicle and arcuate fascicle (SLF/AF; green), and the frontal aslant tract (FAT; blue) **(D)**.

For nrTMS-based DTI FT of language-eloquent white matter pathways we used our standard deterministic algorithm with a fiber assignment by continuous tracking (FACT) (iPlanNet Cranial 3.0.1, Brainlab AG, Munich, Germany). The DTI FTs were performed according to our standard protocol (18, 20, 25). The DTI FTs at PRE-1 and PRE-2 were performed by the use of nrTMS language mapping results and the according DTI sequences at PRE-1 and PRE-2. The DTI FT at POST-1 was performed by the use of nrTMS language mapping results at PRE-1 and the DTI sequences at POST-1. First, we performed a FT of all subcortical pathways connecting language-positive cortical sites in terms of nrTMS (Figure 1). Based on the resulting whole-language-network tractography we added further ROIs in order to track single pathways. For the tracking of the IFOF we set an additional ROI within dorso-rostrally oriented fibers of the external capsule. The SLF/AF was tracked by adding an additional ROI within dorso-rostrally oriented fibers lateral to the posterior horn of the lateral ventricle. For the tracking of the FAT we set an additional ROI within cranio-caudally oriented fibers connecting the superior frontal gyrus and the inferior frontal gyrus (Figure 2). Thereby, we were able to separately visualize single pathways, which were exclusively connecting language-positive cortical sites in terms of nrTMS and additionally passing through one of the three additional ROIs (Figures 1, 2).

#### Data Analysis

In order to correlate the FT results at PRE-1, POST-1, and PRE-2 with the according status of language function, we analyzed the number of fibers for the purpose of a volumetric analysis as calculated by the fiber tracking software (iPlanNet Cranial 3.0.1, Brainlab AG, Munich, Germany). Therefore, we calculated the absolute and relative differences for each pathway at the three examinations. This was done separately for all patients and summarized for the following groups: patients with permanent surgery-related language deficits (= new postoperative language deficit at POD5-1 and POM3-1), patients with transient surgery-related language deficits (= new postoperative language deficit at POD5-1, no language deficit at POM3-1), patients with new surgery-related language deficits, patients with new tumor-related language deficits (= no language deficit at POM3-1, new language deficit at PRE-2 and tumor recurrence in MRI scan), and patients without new language deficits.

## Results

### Patient and Tumor Characteristics

We included 10 patients with a mean age of 50 ± 13.9 years. Table 1 shows detailed patient characteristics of all included patients including the intervals between the different MR images, the extent of resection (EOR), and the detection of subcortical ischemia after the first surgery. Furthermore, Table 1 gives information about the status of language function at each examination during the evaluation period and the assignment of each patient to the different subgroups “permanent surgery-related language deficits,” “transient surgery-related language deficits,” “new tumor-related language deficits,” and “without new language deficit” (Table 1).

**Table 1:** Patient characteristics.

**Patient**	**Age**	**PRE-1 to PRE-2 (months)**	**PRE-1 to 2nd DTI (days)**	**PRE-1 to 3rd DTI (months)**	**Subcortical ischemia after surgery 1**	**EOR**	**Aphasia grading**						**Course of language function**
							**PRE-1**	**POD5-1**	**POM3-1**	**PRE-2**	**POD5-2**	**POM3-2**
1	45	12	15	6	N	GTR	0	1A	1A	1A	–	–	Permanent surgery-related deficit
2	34	26	7	26	Y	GTR	0	1A	0	0	0	0	Transient surgery-related deficit
3	49	37	189	25	Y	GTR	0	1B	0	0	–	–	Transient surgery-related deficit
4	74	2	57	2	N	GTR	0	2A	0	0	–	–	Transient surgery-related deficit
5	31	15	1	14	N	GTR	1B	1B	0	1A	1A	1A	New tumor-related deficit
6	51	14	3	14	N	GTR	0	0	0	1B	1B	1B	New tumor-related deficit
7	56	3	5	3	N	GTR	0	0	0	1A	2A	1A	New tumor-related deficit
8	52	21	120	22	N	GTR	2B	1B	0	0	0	2A	Without new deficit
9	36	30	13	28	N	GTR	0	0	0	0	–	–	Without new deficit
10	72	13	7	13	N	GTR	0	0	0	0	0	0	Without new deficit

*The table shows detailed patient characteristics of all included patients including the intervals between the different MR images, the status of language function at each examination, and the assignment of patients to different subgroups (Y, yes, N, no; EOR, extend of resection, GTR, gross total resection)*.

Table 2 shows detailed information about the tumor type of each patient including WHO grading, IDH mutation, and 1p19q codeletion. Moreover, the table gives information regarding the location of the tumor and if more than 50% of the tumor volume were located subcortically (Table 2).

**Table 2:** Tumor characteristics.

**Patient**	**Surgery before PRE-1**	**Tumor**						**Recurrence at PRE-2**
		**Entity**	**WHO grade**	**IDH mutation**	**1p19q codeletion**	**Location**	**Subcortical**
1	N	GBM	IV	N	–	aSMG	N	N
2	N	AA	III	Y	N	insular	Y	Y
3	Y	DA	II	Y	N	insular	Y	N
4	N	GBM	IV	N	-	mMFG/pMFG	Y	Y
5	Y	AA	III	Y	N	pMFG/opIFG	Y	Y
6	Y	AA	III	N	N	pMTG	N	Y
7	N	GBM	IV	N	–	aSMG	N	Y
8	N	GBM	IV	–	–	STG	Y	Y
9	Y	AA	III	Y	N	pMFG/opIFG	Y	N
10	N	GBM	IV	N	–	STG	Y	Y

*The table shows the tumor characteristics of all included patients and the detailed location of each tumor (Y, yes, N, no; DA, diffuse astrocytoma, AA, anaplastic astrocytoma, GBM, glioblastoma; aSMG, anterior supramarginal gyrus, mMFG, middle middle frontal gyrus; pMFG, posterior middle frontal gyrus; opIFG, opercular inferior frontal gyrus; pMTG, posterior middle temporal gyrus; STG, superior temporal gyrus)*.

### Fiber Tracking Analysis

#### Single White Matter Pathway Analysis

Table 3 shows the absolute number of fibers for each of the separately tracked subcortical pathways at PRE-1, and the percentage changes of nrTMS-based DTI FTs between PRE-1 and POST-1, POST-1, and PRE-2, and PRE-1, and PRE-2 (Table 3). The changes of nrTMS-based DTI FTs of single white matter pathways correlated with the according status of language function for any of the pathways in 80% of patients and in 19 of 30 (63%) single pathway comparisons between PRE-1 and POST-1. Between POST-1 and PRE-2 the nrTMS-based DTI FTs correlated with the status of language function for any of the pathways in all patients and in 24 of 30 (80%) single pathway comparisons. Single FT results correlated with the according status of language function at POST-1 in 60%, 70%, and 60% of cases, and with the according status of language function at PRE-2 in 60%, 90%, and 90% of cases for the tracking of the IFOF, FAT, and SLF/AF, respectively (Table 3).

**Table 3:** Changes of single white matter pathways and correlation with course of language function.

**Patient**	**Number of fibers at PRE-1**				**AG**	**% Difference between PRE-1 and POST-1**				**LF**	**% Difference between POST-1 and PRE-2**				**LF**	**% Difference between PRE-1 and PRE-2**				**LF**
	**IFOF**	**FAT**	**SLF/AF**	**Total**		**IFOF**	**FAT**	**SLF/AF**	**Total**		**IFOF**	**FAT**	**SLF/AF**	**Total**		**IFOF**	**FAT**	**SLF/AF**	**Total**
1	567	2890	3934	7391	0	−85.9	−83.1	−67.5	−75.0	W	1866.3	−22.9	−36.7	49.4	U	177.4	−87.0	−79.4	−62.7	W
2	833	483	2982	4298	0	−37.2	−93.2	−78.8	−72.4	W	−99.0	648.5	29.4	−9.9	I	−99.4	−48.9	−72.6	−75.1	U
3	392	86	274	752	0	−66.8	−100	−26.6	−56.0	W	0.8	–	102.5	76.1	I	−66.6	−47.7	48.5	−22.5	U
4	124	3	4223	4350	0	−98.4	−100	−99.4	−99.4	W	1700.0	–	2215.4	2192.9	I	−71.0	33.3	−85.7	−85.2	U
5	51	0	38	89	1B	−23.5	–	189.5	82.0	U	1564.1	−69.2	−100	303.1	W	1172.5	–	−100	633.7	W
6	2150	6	8499	10655	0	−78.9	−100	−95.9	−92.4	U	−96.3	–	−98.3	−97.1	W	−99.2	−100	−99.9	−99.8	W
7	8747	1522	6588	16857	0	−86.7	−61.8	−97.3	−88.6	U	−99.5	−100	−84.8	−98.3	W	−99.9	−100	−99.6	−99.8	W
8	416	0	2231	2647	2B	−98.3	–	−17.3	−23.6	U	14471.4	−43.9	49.8	91.8	U	145.2	–	23.8	46.5	U
9	109	110	682	901	0	138.5	−100	−76.7	−53.5	U	573.1	–	1059.7	758.0	U	1505.5	−99.1	170.4	299.0	U
10	201	0	62	263	0	613.4	–	7466.1	2228.9	U	−85.5	–	−41.0	−43.3	U	3.5	–	4366.1	1220.5	U

*The table shows the absolute number of fibers for each of the separately tracked subcortical pathways at PRE-1, the initial aphasia grading at PRE-1, and the percentage changes of nrTMS-based DTI FTs between PRE-1 and POST-1, POST-1, and PRE-2, and PRE-1, and PRE-2 as well as the changes of language function in all patients. The table also shows the correlation of the course of language function in each patient and the according difference of fibers of each single white matter pathway. Percentages highlighted in green indicate a correlation of the percentage changes of single white matter pathways and the according course of language function. Percentages highlighted in red indicate a lack correlation of the percentage changes of single white matter pathways and the according course of language function (AF, aphasia grading at PRE-1, LF, language function; W, worsened; U, unchanged; I, improved)*.

#### Subgroup Analysis

**Figure 3:**
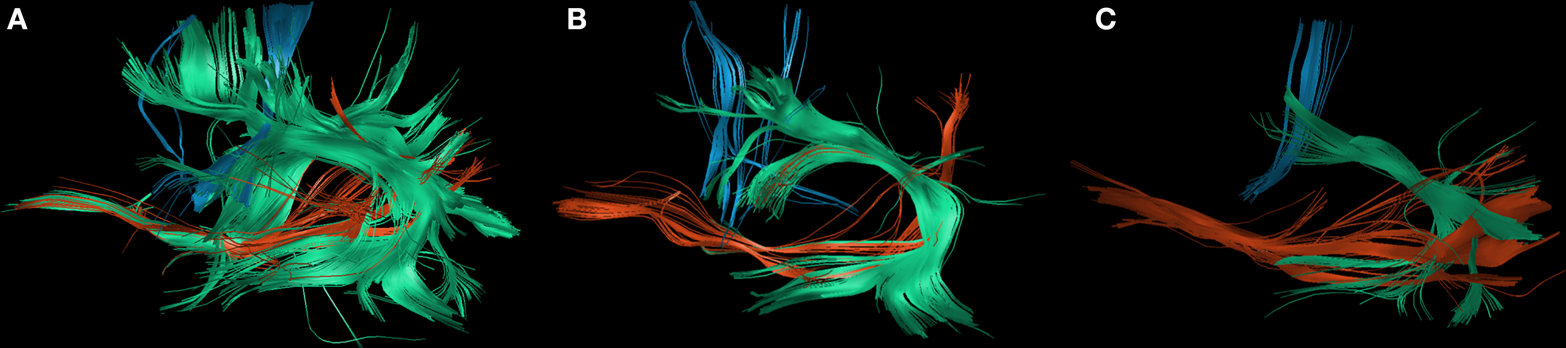
Changes of white matter pathways after permanent surgery-related deficit. The figure shows the changes of the inferior fronto-occipital fascicle (IFOF; red), the superior longitudinal fascicle and arcuate fascicle (SLF/AF; green), and the frontal aslant tract (FAT; blue) at PRE-1 **(A)**, POST-1 **(B)**, and PRE-2 **(C)** in patient 1 who suffered from a permanent surgery-related language deficit grade 1A.

**Figure 4:**
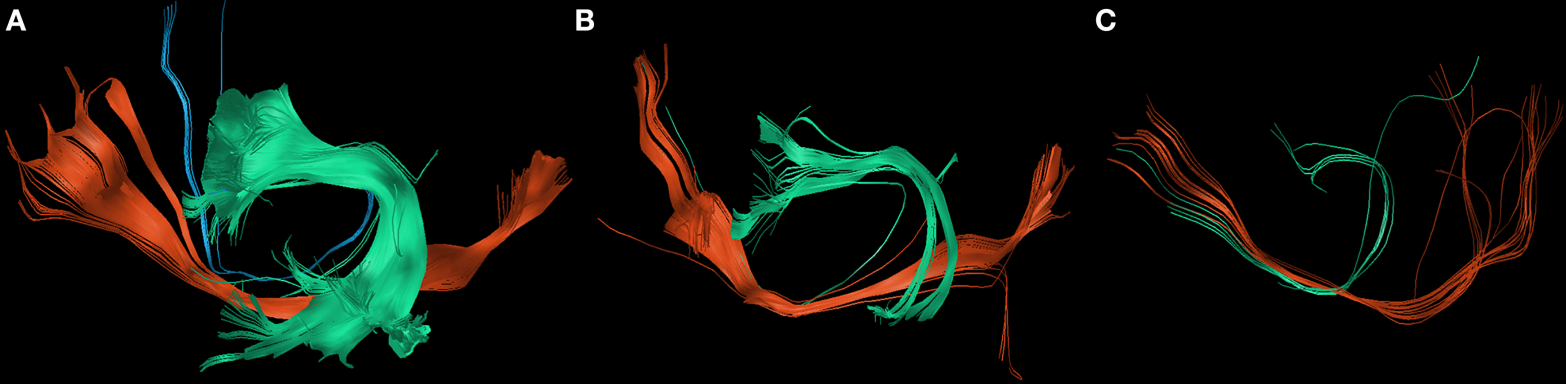
Changes of white matter pathways after tumor-related deficit. The figure shows the changes of the inferior fronto-occipital fascicle (IFOF; red), the superior longitudinal fascicle and arcuate fascicle (SLF/AF; green), and the frontal aslant tract (FAT; blue) at PRE-1 **(A)**, POST-1 **(B)**, and PRE-2 **(C)** in patient 6 who suffered from a new tumor-related language deficit grade 1B.

Postoperatively, we found an overall loss of fibers of the IFOF, FAT, and SLF/AF of −79.8% between PRE-1 and POST-1 in patients suffering from new surgery-related language deficits. Three patients with transient surgery-related language deficits appropriately showed an overall gain of fibers of 48.4% between POST-1 and PRE-2. In contrast, one patient suffering from a permanent surgery-related language deficit also showed an overall gain of fibers of 49.4% depending on a distinct gain of fibers within the IFOF, while fibers within the FAT and the SLF/AF stayed low (Figure 3). Patients with new tumor-related language deficits at PRE-2 showed an overall loss of fibers of −75.5% between POST-1 and PRE-2 (Figure 4). These patients also showed an overall loss of fibers between PRE-1 and POST-1. Three patients without new language deficits showed an overall gain of fibers of 124.8% between PRE-1 and POST-1, and an overall gain of fibers of 27.8% between POST-1 and PRE-2 (Table 4).

**Table 4:** Subgroup analysis.

	**IFOF**	**FAT**	**SLF/AF**	**Total**
**DIFFERENCE BETWEEN PRE-1 AND POST-1 IN NEW**				
**SURGERY-RELATED LANGUAGE DEFICITS**				
Relative	−61.6%	−84.9%	−81.3%	−79.8%
Absolute	−1181	−2940	−9275	−13,396
Mean	−295.3	−735.0	−2,318.8	−3,349.0
SD	130.5	976.4	1,473.5	1,896.8
**DIFFERENCE BETWEEN POST-1 AND PRE-2 IN PERMANENT**				
**SURGERY-RELATED LANGUAGE DEFICIT**				
Relative	1,866.3%	−22.9%	−36.7%	49.4%
Absolute	1493	−112	−469	912
**DIFFERENCE BETWEEN POST-1 AND PRE-2 IN TRANSIENT**				
**SURGERY-RELATED LANGUAGE DEFICITS**				
Relative	−73.7%	797.0%	112.7%	48.4%
Absolute	−483	263	968	748
Mean	−161.0	87.7	322.7	249.3
SD	252.8	90.9	179.3	298.8
**DIFFERENCE BETWEEN PRE-1 AND POST-1 IN NEW**				
**TUMOR-RELATED LANGUAGE DEFICITS**				
Relative	−84.9%	−61.1%	−95.8%	−89.5%
Absolute	−9,290	−933	−14,486	−24,709
Mean	−3,096.7	−311.0	−4,828.7	−8,236.3
SD	3,245.3	444.8	4,332.2	6,231.1
**DIFFERENCE BETWEEN POST-1 AND PRE-2 IN NEW**				
**TUMOR-RELATED LANGUAGE DEFICITS**				
Relative	−59.5%	−99.3%	−94.8%	−75.5%
Absolute	−986	−591	−606	−2,183
Mean	−328.7	−197.0	−202.0	−727.7
SD	726.2	272.3	102.5	973.6
**DIFFERENCE BETWEEN PRE-1 AND POST-1 IN PATIENTS**				
**WITHOUT NEW LANGUAGE DEFICIT**				
Relative	134.3%	55.5%	125.0%	124.8%
Absolute	975	61	3,719	4,755
Mean	325.0	20.3	1239.7	1,585.0
SD	681.5	115.6	2,397.3	3,024.9
**DIFFERENCE BETWEEN POST-1 AND PRE-2 IN PATIENTS**				
**WITHOUT NEW LANGUAGE DEFICIT**				
Relative	75.1%	246.8%	10.2%	27.8%
Absolute	1,277	422	682	2,381
Mean	425.7	140.7	227.3	793.7
SD	1,184.0	253.2	1,551.6	2,495.2

*The table shows the summarized changes of single white matter pathway fibers for the different subgroups of patients*.

## Discussion

### Correlation of Language Pathways With the Course of Language Function

By the present results we were able to show that nrTMS-based DTI FT of the IFOF, FAT, and SLF/AF mainly correlates with the according status of language function preoperatively, postoperatively, and at long-term follow up. Especially new language deficits could be visualized with a high reliability (Figures 3, 4 and Tables 3, 4). In these cases, we could also show a high correlation of the kind of language deficit and the according pathway. In patients suffering from new non-fluent aphasias, we found a loss of SLF/AF and FAT fibers, which are both responsible for phonological processing, articulation, and articulatory planning (Figure 3). In contrast, patients with new fluent aphasias showed a distinct loss of IFOF fibers, which are responsible for semantic information (Tables 1, 3) (8, 9).

The tractography of single white matter pathways particularly correlated for the comparisons between POST-1 and PRE-2. In patients suffering from new surgery-related language deficits we could find a loss of fibers at POST-1 and a gain of fibers at PRE-2 in case of transient language deficits. Although we found an overall gain of fibers for the comparison between POST-1 and PRE-2 in one patient suffering from a permanent language deficit, we still could find less fibers at PRE-2 in comparison with PRE-1 (Figure 3, Table 3). The total gain of fibers at PRE-2 in this patient was due to an increase of IFOF fibers while FAT and SLF/AF fibers were still decreasing. Conveniently, this patient suffered from a permanent non-fluent language deficit. Again, the tractography result of single tracts correlated well with the corresponding language function and kind of deficit as described in literature (9, 30). Patients with new tumor-related language deficits showed an overall loss of fibers at PRE-2, however, these patients already showed an overall loss of fibers at POST-1. In contrast, we could not find a loss of fibers at POST-1 or PRE-2 in patients without any language deficit (Table 4).

### Structural Lesions and Compensatory Mechanisms

As we have learned from DES studies providing resection probability maps, large parts of the cortex are resectable, but lesions within the subcortical white matter pathways cause permanent language deficits in most cases (2, 3). However, this fact does not rule out the possibility of functional reorganization and compensatory mechanisms on a subcortical level. That functional reorganization is able to compensate functional deficits cortically by various mechanisms has already been shown by former studies (16, 30–32). Hereby, the auto-regulation of synapses and mechanisms for the unmasking of latent networks play a major role (33–35). That the human subcortical white matter *per se* harbors multipotential neural progenitor cells has already been proven (36). However, we cannot be sure whether damage to white matter pathways leads to functional reorganization on a subcortical level by rewiring, or whether subcortical changes are only a passive reaction on cortical mechanisms. Not least the results of the present study show that the whole network is able to compensate lesions by the recruitment of accessory and parallel long-distance association pathways and the unmasking of perilesional latent parallel networks (37). These mechanisms might be a reason for the appropriate visualization of a gain of fibers between POST-1 and PRE-2 in patients suffering from transient surgery-related language deficits.

Nevertheless, the impact of surgically induced deficits happens to fast for compensatory mechanisms. Our cohort includes four patients with new surgery-related language deficits and we could find a loss of fibers between PRE-1 and POST-1 for each of the visualized pathways and in all of these 4 patients. However, we could find subcortical ischemia in postoperative MR images in only two cases. Thus, damage to white matter pathways seems not to be an essential reason for subcortical changes. These changes might originate from the cortex. Since these two patients suffered from transient language deficits, the recovery of unimpaired language function as measured at POM3-1 might be due to functional reorganization on the subcortical level. In two patients with new surgery-related language deficits without subcortical ischemia we were also able to find a loss of fibers. In these cases, the loss of fibers must have been caused by the loss of connectivity without a structural lesion on the subcortical level. This hypothesis is supported by the fact that we used the same ROIs of language-positive cortical sites in terms of nrTMS at PRE-1 and POST-1.

We were also able to measure a loss of fibers in 3 patients suffering from new tumor-related language deficits at PRE-2. In contrast, we could not find a loss of fibers in another four patients who also showed a tumor recurrence at PRE-2 without causing a language deficit. Thereby, in case of tumor-related language deficits it has to be assumed that the lack of fibers is caused by an impairment of the whole network. Here, compensatory mechanisms on a cortical and subcortical level seem to be successful regarding the preservation of language function only in some cases. Furthermore, patients with transient surgery-related language deficits after the improvement of their function and patients without any deterioration of language function after the resection of the compromising glioma even showed a gain of fibers.

### Factors Impairing the Reliability of DTI FT

In the present analysis the comparisons of single white matter language pathways correlated with the according status of language function in 63% at POST-1 and 80% at PRE-2, respectively. When analyzing the mismatch of fibers and the status of language function more detailed, we can disregard the mismatch of IFOF fibers at PRE-2 in case 1, 2, and 5. These patients suffered from non-fluent aphasias but IFOF fibers transfer semantic information predominantly. After this correction the mismatch of fibers and status of language function at PRE-2 is only 10%.

In contrast, the reliability of nrTMS-based DTI FT seems to be more impaired directly after surgery. In patients 5, 6, and 7, who did not suffer from a new language deficit at POST-1, we found the highest mismatch of fibers and the according status of language function. Characteristically, these patients had the shortest intervals between PRE-1 and the second DTI imaging at POST-1. Hence, it must be assumed that postoperative edema impairs DTI imaging, even if it does not cause a language deficit. Furthermore, this hypothesis is supported by the results of patients 1–4. These patients suffered from new surgery-related language deficits and their status of language function correlated with nrTMS-based DTI FT results in all cases and for each white matter pathway. Significantly, the intervals between PRE-1 and POST-1 were longer in these cases.

### Future Applications of the Presented Approach

For the present study we used DTI FT as a research tool. We also perform preoperative nrTMS-based DTI FT for motor and language pathways standardly at our department and transfer these data to neuronavigation for the intraoperative illustration (38). Although DTI FT has repeatedly been compared to the gold standard technique of intraoperative subcortical stimulation showing promising results (4, 39–41), it might still serve as a research tool and not a clinical tool (42). In order to preserve function during subcortical glioma resection, the limitations of the underlying techniques *per se* have to be kept in mind. These interdict the exclusive application of preoperative DTI FT data in neurosurgical cases, especially the possibility of revealing false negative results. Methodological innovations for the correction of DTI distortions and new algorithms for the optimization of image fusion address some of these issues. However, the presented approach of nrTMS-based DTI FT enables to give undirected DTI data a function-based starting and endpoint by adding ROIs based on a noninvasive mapping technique based on the same principle as the gold standard technique DES. Thereby, nrTMS-based DTI FT qualifies for long-term follow up examinations of cortical and subcortical functions and might contribute to the decision of re-operation, particularly in patients suffering from tumor residuals or recurrence of highly eloquent low grade gliomas. Additionally, the presented technique of single white matter pathway tracking might help basic researchers to assign higher brain functions to specific white matter pathways and subcortical networks.

### Limitations

For the present study we used a deterministic DTI tractography algorithm. Pros and cons of deterministic vs. probabilistic algorithms and different diffusion models such as q-ball imaging and multiple others are controversially discussed (43, 44). Comparisons between different algorithms and imaging techniques showed that the tractography result is highly dependent on the respective mode (45–47). Hence, the application of only one tractography algorithm must be seen as a limitation of our study. In contrast, by using ROIs based on the preoperative mapping by nrTMS, the present study outlines the beneficial option of giving tractography results a function-based starting and end point. Our presented standard protocol is integrated in the setup of our department. Here it must be highlighted that the feasibility with regard to the time that is required for the performance of tractography must be considered especially for its application in neurosurgery. Furthermore, the core of our results bases on relative changes of white matter language pathways and the correlation to the according status of language function. Although other tractography algorithms might have revealed different total numbers of fibers, the analysis of relations in the present study rules out a fundamental misinterpretation of data.

Changes in a functional network, particularly after the resection of gliomas, always base on a combination of cortical and subcortical adaptations. As shown by cortical language re-mappings by direct cortical stimulation during awake craniotomy, the cortical localization of language function changes over time after the resection of gliomas (32). However, for the present manuscript we focused on subcortical changes in dependence on the current status of language function. Particularly permanent language deficits after the resection of gliomas are mainly due to damaged white matter pathways. Based on the present results we were able to confirm this by DTI FT of single white matter pathways. We can exclude the impact of cortical changes between PRE-1 and POST-1 since we used the same cortical ROIs for these two DTI FTs. For the DTI FT at PRE-2 we used new nrTMS language mapping results at PRE-2 and the according DTI sequences at PRE-2 as described in the methods section. Cortical nrTMS language mapping data were used for the initial whole-language-network tractography. By the setting of additional anatomical ROIs for the single white matter pathway tracking of the FAT, the IFOF, and the SLF/AF, the algorithm is able to visualize definitely existing fibers exclusively. Thereby, the influence of cortical changes between these two examinations is reduced to a minimum through the special technique of single white matter pathway trackings we used for the present analysis.

Finally, the cohort size of our pilot study with only 10 patients and the variability of time intervals between imagings and mappings is a limitation of our study. However, the present cohort enabled us to provide highly valuable tractographies of white matter language pathways in glioma patients including long-term follow up data and the positive correlation with the according status of language function.

## Conclusion

By the present results we were able to show that nrTMS-based DTI FT of the IFOF, FAT, and SLF/AF mainly correlates with the according status of language function preoperatively, postoperatively, and at long-term follow up after the resection of left-sided perisylvian gliomas.

## Author Contributions

SI is responsible for data acquisition and handled the acquired data, performed statistical analyses, performed literature research, and drafted the manuscript. LE was responsible for data acquisition. AK was responsible for data acquisition. BM approved and corrected the final version of the manuscript. SK revised the manuscript, approved and corrected the final version, and is responsible for the original idea, the concept, design, data acquisition, and statistical analyses. All authors read and approved the final manuscript.

### Conflict of Interest Statement

BM received honoraria, consulting fees, and research grants from Medtronic (Meerbusch, Germany), Icotec ag (Altstätten, Switzerland), and Relievant Medsystems Inc., (Sunnyvale, CA, USA), honoraria, and research grants from Ulrich Medical (Ulm, Germany), honoraria and consulting fees from Spineart Deutschland GmbH (Frankfurt, Germany) and DePuy Synthes (West Chester, PA, USA), and royalties from Spineart Deutschland GmbH (Frankfurt, Germany). SK is consultant for Nexstim Plc (Helsinki, Finland) and Spineart Deutschland GmbH (Frankfurt, Germany) and received honoraria from Medtronic (Meerbusch, Germany) and Carl Zeiss Meditec (Oberkochen, Germany). SK and BM received research grants and are consultants for Brainlab AG (Munich, Germany).

The remaining authors declare that the research was conducted in the absence of any commercial or financial relationships that could be construed as a potential conflict of interest.

## Abbreviations

AAT: Aachener Aphasia Test
AF: Arcuate fascicle
DES: Direct electrical stimulation
DTI FT: Diffusion tensor imaging fiber tracking
EC: External capsule
FAT: Frontal aslant tract
FACT: Fiber assignment by continuous tracking
IFOF: Inferior fronto-occipital fascicle
ILF: Inferior longitudinal fascicle
nrTMS: Navigated repetitive transcranial magnetic stimulation
ON: Object naming task
POD5-1/-2: 5 days after surgery 1/2
POM3-1/-2: 3 months after surgery 1/2
POST-1: Between PRE-1 and PRE-2
PRE-1/-2: Before surgery 1/2
ROI: Region of interest
SLF: Superior longitudinal fascicle
SMA: Supplementary motor areas
UF: Uncinate fascicle.
